# Imaging in clinical trials of axial spondyloarthritis: what type of imaging should be used

**DOI:** 10.1007/s00256-025-04935-0

**Published:** 2025-05-10

**Authors:** Iris Eshed, Kay Geert A. Hermann

**Affiliations:** 1 Sheba Medical Center, Department of Radiology, Tel Hashomer, affiliated to the Tel Aviv University School of Medicine, Tel Aviv, Israel; 2 Department of Radiology, Charité – Universitätsmedizin Berlin, Charitéplatz 1, Berlin, 10117 Germany

**Keywords:** Arthritis, Spondyloarthritis, MRI, Radiography, Scoring systems, Imaging as biomarker

## Abstract

Axial spondyloarthritis (axSpA) is a chronic inflammatory condition predominantly affecting the sacroiliac joints and spine. Early and accurate diagnosis is crucial to prevent structural damage and improve patient outcomes. Imaging plays a pivotal role in axSpA diagnosis, monitoring, and clinical trials, offering insights into both inflammatory activity and structural progression. Conventional radiography has been foundational for detecting structural changes, such as syndesmophytes and erosions, but it is limited by poor sensitivity for early disease detection and significant interobserver variability. Advanced imaging modalities, such as magnetic resonance imaging (MRI) and low-dose computed tomography (ld-CT), have emerged as more sensitive tools. MRI excels in identifying active inflammation, particularly bone marrow edema, and is integral to early diagnosis and disease monitoring. ld-CT provides superior spatial resolution for detecting structural lesions while minimizing radiation exposure. However, challenges remain in achieving standardized imaging protocols and consistent scoring systems across clinical trials. Scoring systems like the modified Stoke Ankylosing Spondylitis Spine Score (mSASSS), Spondyloarthritis Research Consortium of Canada (SPARCC) scores, and Berlin methods require rigorous calibration to ensure reliability. The purpose of this review is to explore the strengths and limitations as well as the use in clinical trials of the different imaging modalities and to offer guidance on selecting the most suitable imaging techniques for assessing both disease activity and structural progression in clinical trials.

## Introduction

Axial spondyloarthritis (axSpA) is a chronic inflammatory disease predominantly affecting the sacroiliac joints (SIJs) and the spine [1]. The burden of disease in axSpA is substantial, not only due to chronic pain and reduced quality of life but also because of the potential for long-term structural damage, leading to functional impairment and disability [2]. Early diagnosis and intervention are crucial for preventing irreversible structural damage and for improving patient outcomes [3].

Imaging plays a central role in both the diagnosis and monitoring of axSpA. It is indispensable for evaluating disease activity, particularly inflammation in the SIJs and spine, and for assessing structural progression over time [4, 5]. While conventional radiography has long been used to identify structural long-term changes, such as syndesmophytes and erosions on the SIJs [6, 7], more advanced imaging modalities like magnetic resonance imaging (MRI) and low-dose computed tomography (ld-CT) are proving to be more sensitive for detecting inflammation and early structural lesions, respectively [8, 9]. MRI, in particular, has gained prominence as the modality of choice for detecting active inflammation, even before structural damage becomes evident on radiographs [4]. Additionally, it offers valuable insights into bone marrow edema (BME), a hallmark of active disease, which has significant implications for both diagnosis and treatment response [10, 11].

Given the critical role of imaging in axSpA, its application in clinical trials is of paramount importance. In clinical trials, accurate and sensitive imaging modalities are essential for assessing disease activity, monitoring response to therapy, and tracking structural progression [12]. Furthermore, imaging can serve as a key endpoint in trials, providing objective measures of both inflammatory and structural changes [12]. However, the choice of imaging modality in clinical trials is not straightforward, as it must balance sensitivity, specificity, practicality, and safety considerations, including radiation exposure in long-term studies.

The purpose of this review is to explore the strengths and limitations as well as the use in clinical trials of the different imaging modalities and to offer guidance on selecting the most suitable imaging techniques for assessing both disease activity and structural progression in clinical trials.

## Imaging modalities and associated scoring systems in axial spondyloarthritis

### Conventional radiography

Inflammation of the SIJs and the spine leads to destruction of the bony cortex and new bone formation, which can be detected on conventional radiographs of the SIJs and spine [13]. Thus radiographs have been employed for diagnosing and assessing ankylosing spondylitis and other forms of SpA since the 1930 s, and they have remained a standard component of the diagnostic evaluation for patients with suspected SpA for many years [14, 15]. While structural lesions, which manifest as erosions, periarticular or corner sclerosis, syndesmophytes, and ankylosis, may take years to develop following the onset of inflammation [16], inflammation itself may be readily seen on MRI [17], proving radiographs an inappropriate modality for early detection of axSpA.

In addition, interpreting pelvic radiographs, especially of the SIJs, is challenging even for experienced radiologists due to the limitations of interpreting a two-dimensional image of a three-dimensional structure. Furthermore, the SIJ contour is often obscured by superimposed bowel gas or feces, and the oblique orientation of the sacral and iliac portions results in overlapping images that can mimic structural lesions of sacroiliitis, complicating reliable evaluation. A major drawback of radiographic SIJ assessment is the significant interobserver and intraobserver variability in sacroiliitis evaluation, as demonstrated in a large-scale study where substantial interobserver variation was observed, particularly in early-stage sacroiliitis [18, 19]. Sensitivities and specificities for radiographic sacroiliitis were moderate, with high rates of false positives and negatives, and neither individual nor group training improved accuracy [19]. Another study also found low concordance among readers, with erosion identified as a key source of disagreement [20]. These findings suggest that SIJ radiographs are generally unreliable for diagnosing and grading sacroiliitis, particularly in early stages.

Structural lesions in the spine, particularly syndesmophytes, are not included in the current classification or diagnostic criteria for axSpA because the disease typically begins in the SIJs. The occurrence of spinal syndesmophytes in the presence of radiologically normal SIJs is uncommon, though possible. Radiographs of the thoracic spine, the spinal segment most commonly affected in axSpA [21], are often limited by the superimposition of the heart and mediastinal structures as well as ribs, which hampers reliable evaluation of post-inflammatory structural lesions. Still syndesmophytes are more readily detected on spinal radiographs compared to MRI [22] and thus cervical and lumbar spine radiographs are considered cornerstone in the evaluation of syndesmophytes in axSpA and are complementary to MRI in the evaluation of structural lesions [21].

### Scoring systems using X-rays

Radiographic damage is a key outcome measure in axSpA and it was shown to be associated with reduced spinal mobility and functional disability over time [23]. The most commonly used scoring systems for assessing radiographic damage in axSpA are the 1984 modified New-York criteria (mNYC) [24] for the evaluation of SIJs and the modified Stoke Ankylosing Spondylitis Spine Score (mSASSS) for the evaluation of the spine [25].

#### Modified New York Criteria

Imaging-based confirmation of sacroiliitis enhances the specificity of classification criteria compared to relying solely on clinical evaluation [26]. Consequently, advanced radiographic sacroiliitis was incorporated as one of the three classification criteria for ankylosing spondylitis in the mNYC [24]. The mNYC introduced a threshold for radiographic sacroiliitis to enhance specificity and interrater reliability in interpreting the findings [24]. This threshold is based on a 5-point grading scale for both sacroiliac joints. Radiographic evidence of bilateral grade 2 or unilateral or bilateral grade 3 or 4 is required to meet the diagnostic threshold for sacroiliitis [24].

However, because radiographic changes in axSpA develop slowly and may not be detectable in the early stages of the disease, the mNYC have low sensitivity for identifying early sacroiliitis on radiographs [27].

The mNYC are often used as an entry filter for axSpA clinical trial helping ensure that participants meet specific diagnostic requirements crucial for the validity of the trial results. These include biologic therapy short- and long-term efficacy trials as well as trials that compare treatment outcomes between patients with radiographic vs. non-radiographic axSpA (nr-axSpA). Also, trials examining the impact of switching between different classes of treatments (e.g., from nonsteroidal anti-inflammatory drugs to biologics) often require participants to meet the mNYC criteria for appropriate study population selection.

#### The modified Stoke Ankylosing Spondylitis Spine Score (mSASSS)

The mSASSS is designed to assess structural lesions in the cervical and lumbar spine including erosions, sclerosis, squaring, syndesmophytes, and bone bridges at the anterior vertebral corners [25]. In this scoring system, each vertebral corner is scored from 0 to 3, where 0 indicates normal; 1 represents the presence of erosion, sclerosis, or squaring; 2 indicates syndesmophytes; and 3 denotes complete bony bridging (ankylosis). The total score ranges from 0 to 72, reflecting the severity of structural damage [25]. mSASSS is to date the most sensitive method for evaluating structural progression over 5–10 years of follow-up in the spine [28, 29] and has been included in the Assessment of Spondyloarthritis international Society (ASAS) core outcome set for axSpA as a mandatory instrument for disease-modifying drug trials [30].

### Magnetic resonance imaging

MRI is currently regarded as the most sensitive and accurate tool for diagnosing early SpA, enabling radiation-free, reliable assessment of the two primary disease components: active inflammation and structural damage.

The main acute inflammatory findings of sacroiliitis, observed on fluid-sensitive MRI sequences such as STIR and T2-weighted (T2-W) with fat suppression (FS) sequences, include periarticular SIJ BME, or osteitis [31] which may be seen years before late structural lesions are evident [32]. Other findings detected on fluid-sensitive MRI sequences that have been correlated to acute inflammatory sacroiliitis include enthesitis, capsulitis, and fluid within the SIJs [33, 34]. Structural lesions, believed to result from this periarticular inflammation, are best detected on T1-weighted (T1-W) sequences and include erosions, periarticular and intraarticular (backfill) fat lesions, and ankylosis [33, 34]. On MRI of the spine, similarly acute (BME, enthesitis, synovitis) and structural (erosions, fat lesion, ankylosis) lesions are detected on fluid-sensitive and T1-W sequences, respectively [35, 36].

Using MRI as a diagnostic tool to identify early inflammatory involvement of the SIJs has enabled differentiation between two major groups of axSpA: nr-axSpA, where patients have normal SIJ radiographs but exhibit acute inflammation on MRI suggestive of sacroiliitis, and radiographic axSpA, where structural lesions suggestive of axSpA are already visible on radiographs [37].

Due to its high sensitivity for early sacroiliitis, MRI of the SIJs was incorporated into the ASAS classification criteria for the inclusion of patients with axSpA into clinical trials [37, 38]. According to the ASAS criteria, a “positive MRI” of the SIJ in patients with axSpA is identified when more than one BME lesion appears on a single MRI slice. If only one BME lesion is present, it must appear on at least two consecutive slices [39]. Of note, BME lesions must be highly suggestive of SpA. The original ASAS definition for sacroiliitis was based solely on the presence of BME, primarily because it is easy to detect and, unlike radiographs, shows high reader agreement and reproducibility [40]. However, in a 2016 update, while BME remained essential for defining sacroiliitis, the presence of structural damage was acknowledged as a contributing factor in the diagnostic process, though it was not required to meet the definition [41].

Spinal findings are not included in the classification criteria for axSpA, primarily because the SIJs are the first site affected in most patients. However, some patients may exhibit spinal involvement even in the absence of SIJ pathology [42]. Due to its high sensitivity to detecting acute and structural lesions, MRI has become the preferred modality in research, both for study entry classification criteria and as an outcome measure of the study’s objectives.

### Scoring systems using MRI

#### Sacroiliac joints

MRI plays a central role not only in assessing inflammatory and structural lesions in the SIJs and in diagnosing and monitoring treatment response but also as a key criterion for patient classification in clinical studies and in evaluating study outcomes. The two main SIJ scoring systems are the Spondyloarthritis Research Consortium of Canada (SPARCC) inflammation [43] and structural scores [44], and the Berlin score [45, 46]. Table 1 summarizes the main features of these two scoring systems. Both scores were shown to be not only reliable and reproducible but also reflect response to treatment and are thus of major importance in clinical trials evaluating novel drugs and therapeutic approaches [47].

**Table 1:** SIJ MRI scoring systems

	SPARCC score	Berlin score
Inflammatory score		
Plane evaluated	Coronal oblique	Coronal oblique
Seq. evaluated	T2 W-FS, fluid sensitive	T2 W-FS, fluid sensitive
Scoring area	8 quadrants **per slice** *(4 quadrants per joint per slice)*	4 quadrants **per joint**
Scoring slices	6 consecutive slices that capture the most inflammation	All slices covering the joint
BME score	0: none, 1: present Per quadrant, *per slice*	0: none, 1: ≤33%, 2: 33–66%, 3: >66% of quadrant
Additional points	Intense:1, depth:1	None
Max. score per slice	12	Not applicable
Max. score per exam.	72	24
Structural score		
Plane evaluated	Coronal oblique	Coronal oblique
Seq. evaluated	T1-W, fat sensitive	T1-W, fat sensitive
Scoring area	8 quadrants **per slice,** for erosion and fat. 4 joint-halves per slice for backfill and ankylosis	4 quadrants **per joint**
Scoring slices	5 consecutive slices in the cartilaginous part	All slices covering joint
Erosion score	0: none, 1: present Per quadrant	0:none, 1: ≤33%, 2: 33–66%, 3: >66% of quadrant
Fat lesion	0: none, 1: present Per quadrant	0: none, 1: ≤33%, 2: 33–66%, 3: >66% of quadrant
Sclerosis	Not usually scored	0: none, 1: present; per joint
Backfill	0: none, 1: present; per joint-half	Not scored
Ankylosis	0: none, 1: present; per joint-half	0: none, 1: present; per joint
Max. score per slice	Erosion and fat = 8 per slice; Backfill and ankylosis = 4 per slice.	Not applicable
Max. score per scoring domain	Erosion: 40 Fat: 40 Backfill: 20 Ankylosis: 20	Erosion: 24 Fat: 24 Sclerosis: 2 Ankylosis: 2

*SPARCC*, SpondyloArthritis Research Consortium of Canada; *Seq.*, sequence; *T2 W-FS*, T2-weighted with fat suppression; *BME*, bone marrow edema; *Max.*, maximum

##### **The SPARCC inflammation and structural scores**

The SPARCC Inflammatory Score is aimed at assessing active inflammation, specifically BME, on semicoronal fluid-sensitive, T2-W MRI sequences. In this score, each SIJ is divided into four quadrants on each MRI slice, and scoring is performed on six consecutive MRI slices that cover the joint. BME is scored binarically (1 = present, 0 = absent) in each quadrant on each slice, with a maximum score of 4 per joint and 8 per slice. Additional points are given if the edema is intense and/or exceeds the depth of the lesion of 1 cm within a slice. The maximum SPARCC inflammatory total score is 72, with higher scores indicating greater inflammatory activity [43].

The SPARCC Structural Score is aimed at assessing chronic, structural lesions in the SIJs on semicoronal fat sensitive, T1-W MRI sequences. Scoring addresses the presence or absence of different structural lesions utilizing a similar quadrant system to the inflammatory score, dividing each SIJ into four quadrants. However, here only five consecutive slices are scored through the cartilaginous portion of the joint. Erosions and fat lesions are scored just like BME as present or absent in each quadrant on each slice with a maximum score of 40. Backfill and ankylosis are scored separately per joint resulting in a maximum score of 20. The SPARCC structural score is cumulative, reflecting the degree of structural progression, with higher scores indicating more extensive chronic changes [44].

##### **The Berlin inflammation and structural scores**

Here too, each SIJ is divided into four quadrants resulting in eight MRI quadrants. The active MRI lesions (mainly BME) are scored on fluid-sensitive T2-W sequences and the structural ones (erosion, sclerosis, fat, ankylosis) are scored on a fat-sensitive, T1-W sequence. The volume of each lesion is graded semiquantitatively for an SIJ quadrant into 0: No BME; 1: < 33%; 2: 33–66%; and 3: > 66%10. Thus, the cumulative total score per patient may reach 24 [45]. A modification of this score, the 24-regions-score, takes into account whether the lesions are located in the anterior, middle, and posterior parts of the joints [48].

**Fig. 1:**
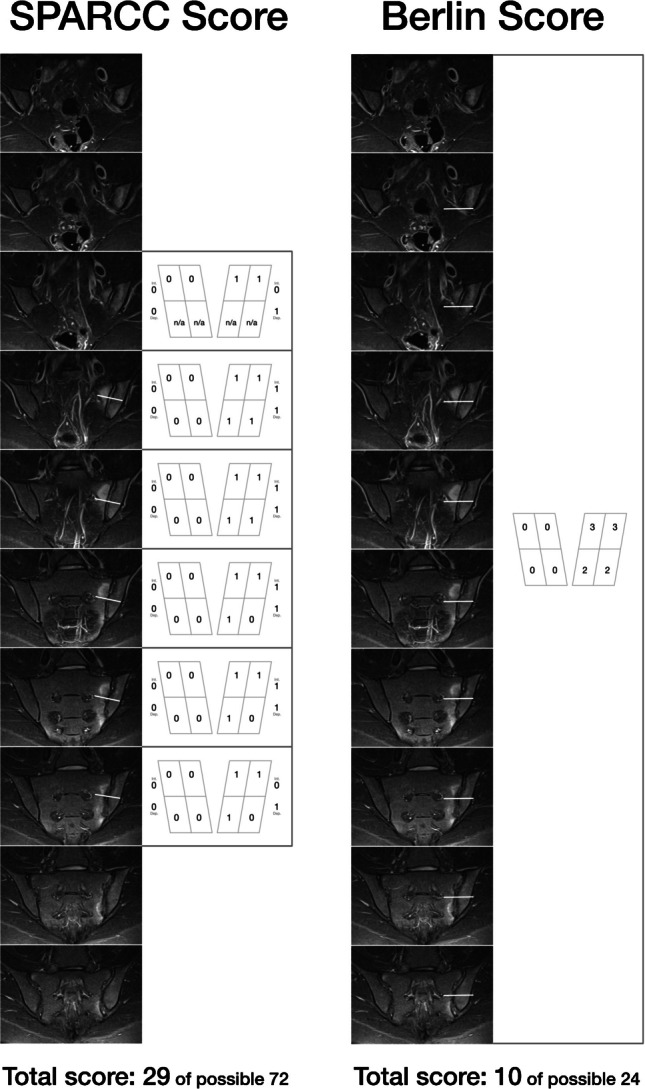
Comparison of SPARCC and Berlin methods: While SPARCC scores the six most inflamed slices in a dichotomous fashion with additional points for signal intensity and size (depth) of the bone marrow edema, the Berlin system uses a global approach by assigning a value between 0 and 3 related to a visual approximation of the volume of the inflamed bone per quadrant. White lines represent the borders between the upper and lower quadrants while the joint space provides an anatomic border between the sacral and iliac quadrants

The typical application of both the Berlin MRI score and the SPARCC MRI score is visualized in Fig. 1.

#### Spine

Similar to the SIJs, spinal MRI is sensitive in detecting acute and structural lesions and has been shown to predict Bath AS Disease Activity Index (BASDAI) response in patients undergoing TNF-α blocker therapy [49]. There are several spinal MRI scoring systems aimed at the quantification of acute and structural lesions for baseline and outcome objective assessment. The main three scoring systems, developed for study purposes, are the AS spinal MRI (ASspiMRI) score [50], the Berlin MRI score [51], and the SpA research Consortium of Canada (SPARCC) MRI score [52]. All three methods demonstrated strong performance in terms of discrimination, feasibility, and responsiveness [53]. The scoring in all three systems use sagittal MRI sequences of the entire spine and refer to vertebral units (VU) defined as the region surrounding an intervertebral disc space including the two endplates and their subchondral bone. However, they differ in many other parameters such as the sequences used to detect inflammation, the number of slices evaluated, the number of vertebral units assessed, and the extent of the subchondral endplate examined. Table 2 summarizes these three scoring methods.

**Table 2:** Spine MRI scoring systems

	ASspiMRI	Berlin score	SPARCC score
Plane evaluated	Sagittal	Sagittal	Sagittal
Seq. evaluated Inflammation			
Structural	T2 W-FS, Fluid sens. T1 W-FS+Gd T1 W	T2 W-FS, Fluid sens. T1 W	T2 W-FS, Fluid sens. T1 W
Scoring area	23 Disco-vertebral units	23 Disco-vertebral units	6 Disco-vertebral units, or 23 Disco-vertebral units^1^
Scoring slices	All slices (scored collectively)	All slices (scored collectively)	3 most severely affected slices (each slice scored individually)
BME score: Additional points:	0–3 per DVU Erosions: 0–3	0–3 per DVU N/A	0–1 per quadrant Intense:1, depth:1
Max score per DVU:	6	3	18
Maximum score (inflammation)	138	69	108 – SPRCC 6-DVU 414 – SPARCC 23-DVU^1^
Structural damage evaluated	Fat lesion, erosion, syndesmophytes, ankylosis (as separate score)	Fat lesions (as separate score)	N/A^2^

*ASspiMRI*, ankylosing spondylitis spinal MRI; *SPARCC*, SpondyloArthritis Research Consortium of Canada; *Seq.*, sequence; *Sens.*, sensitive; *BME*, bone marrow edema; *Max.*, maximum; *DVU*, disco-vertebral unit

^1^Depending on the research question, the SPARCC system is used capturing the six mostly affected DVUs or all 23 DVUs

^2^Structural damage evaluation in the spine is usually done applying the Canada-Denmark (CANDEN) scoring system

##### **AS spinal MRI (ASspiMRI) score**

The score is based on grading of both disease activity and structural damage on a scale of 0 to 6. In this method, all 23 VU of the spine (C2-S1) are scored sagittal T1-W without FS, fluid-sensitive T2-W–FS, and, optionally, T1-W-FS after contrast injection (T1 FS+Gd). VUs are defined as the region between two virtual lines through the middle of each vertebra and are scored in a slice representing the highest level of inflammation in the evaluated VU. Inflammation, evaluated on the T1 FS+Gd or fluid-sensitive sequences, is scored for enhancement or BME in each VU on a scale of 0–3 equivalent for mild (≤25%), moderate (>25% and ≤50%), and severe (>50%) involvement. If erosions are evident of those sequences, a score of 4–6 is given similarly addressing the severity of erosions leading to a maximum score of 138 for the entire 23 VUs. Structural damage is evaluated on a score of 1–6 using a T1-W sequence evaluating a combination of fat lesions, erosions, small and large syndesmophytes, and partial and complete ankylosis.

##### **The Berlin MRI score**

The Berlin score is a modification of the ASspiMRI score with a simpler approach. In this scoring system, BME (irrespective of concomitant erosions) is scored from 0 to 3 for each VU with a maximum whole spine score of 69. Structural lesions are evaluated on a T1-W sequence for the presence of fat lesions (score 0–3) and erosions (score 0–2) per vertebral corner.

##### **The SpA Research Consortium of Canada (SPARCC) MRI score**

Here, the entire spine is evaluated for inflammation, but only the six most severely affected VU are scored according to the original version of this method [52]. Each VU is divided into four quadrants (two anterior and two posterior). For each lesion, three consecutive sagittal slices are assessed. The presence of an increased signal in each of the quadrants on the fluid sensitive sequence is scored binarically (1: presence; 0: absence) and repeated for each of the three consecutive sagittal slices, thus reaching a maximum score per VU of 12. An additional score of 1 per VU is given for increased signal intensity and another for increased signal depth extending more than 1 cm from the endplate thus reaching a maximum SPARCC inflammatory score of 108.

The SPARCC MRI spinal inflammation index has evolved significantly since its development. Initially validated in 2007, the method demonstrated excellent reliability and responsiveness, with analysis limited to the six most affected levels capturing 62% of all affected DVUs and 74% of the total score [54]. However, advancements in imaging workflows, such as PACS systems and digital data entry, have diminished the time-saving advantage of the 6-DVU approach. By 2010, studies comparing the 6-DVU and 23-DVU methods showed equally high interobserver reliability and discriminatory power, with the 23-DVU method favored in observational studies addressing broader research questions [55].

##### **Canada-Denmark MRI scoring system**

Here too the entire spinal 23 VU are evaluated for the presence of a non-bridging or a bridging syndesmophyte. A syndesmophyte is recorded as present or absent at the anterior corner, central segment, and posterior corner locations of each vertebral endplate, and at each facet joint [56].

## Computed tomography

CT is characterized by rapid acquisition, excellent spatial resolution, and superior multiplanar reconstruction capabilities, making it superior to radiographs for detecting structural lesions in the sacroiliac joints [57]. However, due to its high radiation exposure, its use is generally not justified for young patients with axSpA [15]. Consequently, CT is seldom used in the clinical assessment of suspected axSpA, unless there are additional indications for its use [58].

Currently, advances in CT technology enable significant reduction of radiation exposure while maintaining diagnostic accuracy, making low-dose CT (ldCT) feasible for routine clinical applications [59, 60]. Therefore, when confined to the SIJs, the radiation dose can be reduced to under 1 mSv, comparable to that of a single anterior-posterior pelvic radiograph [59, 60]. Similarly, scanning the entire spine with ldCT results in an effective dose of approximately 4 mSv, about half the radiation dose of conventional CT [61, 62]. An even greater dose reduction was reported by using photon counting CT of the spine in which mean effective radiation dose was 1.57 mSv (range 1.11–2.30 mSv) [63].

The anatomy of the SIJ and the spine is clearly demonstrated on CT, allowing for reliable evaluation of structural lesions such as erosions and syndesmophytes which may be challenging to detect on plain radiographs. Indeed, ldCT of the SIJ was reported to be more sensitive for detection of erosions or sclerosis than plain radiography and to be highly specific for axSpA while maintaining high interreader reliability [17, 64]. Likewise, using ldCT of the spine enabled detection of significantly more new and growing syndesmophytes over a 2-year period compared to radiographs [65].

### Sacroiliac joints

Although ldCT shows great potential due to its high sensitivity and specificity in detecting structural lesions, there are currently no validated scoring systems for its application in clinical studies, either as an entry criterion or as an outcome measure.

### Spine

An adaptation of the radiographic scoring of the spine to CT scoring was reported both for mSASSS and for CANDEN scoring systems. In addition, the CT Syndesmophyte Score (CTSS) was specifically developed to be used with ld-CT [61].

### CT Syndesmophyte Score

A semiquantitative scoring system that is analog to the radiographic spinal scoring system mSASSS was developed for the assessment of syndesmophytes on CT and named Computed Tomography Syndesmophyte Score (CTSS) [61, 65]. In this score, 23 VU are evaluated in the sagittal and coronal planes. The presence of a syndesmophyte is assessed in eight quadrants per VU.

A syndesmophyte is scored from 0 to 3 based on its height relative to the intervertebral disc space (IDS), with 0=no syndesmophyte, 1 = <50% of the IDS, 2=≥ 50% of the IDS, and 3=bridging syndesmophyte. This scoring system allows for a maximum possible score of 552 for a patient.

Due to the low sensitivity to change of radiographic mSASSS, at least 2 years is typically required to reliably detect structural progression of the spine [66]. The modified CT mSASSS score was shown more sensitive than radiography for syndesmophyte growth for that same period [65]. However, the proportion of patients with changes beyond the smallest detectable change was similar by ld-CT and radiography.

## Key considerations for selecting imaging modalities in clinical trials

### Sensitivity and specificity of the scoring methods

The selection of imaging modalities in clinical trials depends heavily on their ability to detect both inflammatory and structural changes with high sensitivity and specificity. MRI is regarded as the gold standard for detecting active inflammation, such as BME, due to its exceptional sensitivity and reproducibility [39]. In contrast, ld-CT is superior for detecting structural lesions like syndesmophytes and erosions compared to radiographs, making it particularly valuable for assessing structural progression. Radiographic scoring systems, such as the mSASSS, remain foundational for long-term monitoring of syndesmophyte growth; however, they may lack the sensitivity to detect minor changes over shorter periods, which limits their utility in some clinical trial designs [67].

### Feasibility and practicality

The practicality of imaging modalities in clinical trials is influenced by factors such as availability, cost, and the ease of standardizing scoring systems. Scoring methods are typically selected based on several key factors, including their relevance to the study endpoints, the modality’s ability to detect changes of interest, and their reproducibility across sites and readers. For example, MRI offers a radiation-free option that is especially advantageous for younger populations and studies requiring frequent imaging over long periods. However, its interpretation requires a high level of expertise, and the reproducibility of scores, such as the SPARCC or Berlin scores, relies heavily on rigorous standardization of image acquisition protocols [34] and reader training.

Conversely, ld-CT, with its superior spatial resolution for structural changes, is an increasingly viable alternative [9]. Establishing low-dose protocols suitable for clinical trials necessitates access to modern CT scanners, along with a dedicated team of radiographers, technicians, and radiologists to ensure both safety and accuracy. Cost and logistical considerations, including the availability of equipment and trained personnel, often play a pivotal role in selecting the appropriate imaging modality and scoring system.

### Radiation exposure

Radiation exposure is a critical factor in clinical trial imaging, particularly for longitudinal studies that require repeated assessments. Conventional radiographs and ld-CT used to differ significantly in their radiation doses. While radiographs involve relatively low radiation exposure, their utility is limited by poor sensitivity to early or subtle changes. Advances in ld-CT technology, including photon counting CT, have significantly reduced radiation exposure doses, making them comparable to or even lower than standard radiographic procedures for specific applications [9]. For instance, radiation doses in ld-CT of the SIJ can be reduced to below 1 mSv, aligning with the dose of a single pelvic radiograph [59, 60].

Despite these advancements, ethical concerns about cumulative radiation exposure persist, particularly for younger patients and trials with long follow-up periods. Consequently, MRI remains the preferred modality for studies involving frequent imaging, as it eliminates the risk of radiation exposure entirely while maintaining high sensitivity for both inflammatory and structural changes.

## Challenges and future directions

### Standardization across trial centers

One of the primary challenges in imaging for clinical trials is achieving standardization across multiple trial centers, particularly in international studies. Variability in MRI and CT acquisition protocols can lead to inconsistent image quality, potentially affecting the reliability of assessments. Developing standardized acquisition protocols that can be universally implemented is essential for ensuring consistency [34]. Similarly, harmonizing scoring systems such as SPARCC, Berlin method, and mSASSS across trials is critical for reproducibility and comparability of results. This requires extensive reader training and interreader calibration to minimize variability in interpretations [68, 69].

### Longitudinal imaging

Long-term clinical trials pose unique challenges in utilizing imaging to monitor treatment outcomes. Serial imaging is vital for detecting cumulative damage and assessing response to therapy over time, but ensuring consistency in imaging quality and scoring methods throughout the trial duration is complex. Moreover, defining the clinical relevance of subtle changes observed on imaging remains an ongoing area of investigation [70]. Efforts should focus on identifying thresholds for meaningful changes that correlate with functional outcomes and quality of life [71].

### Cost-effectiveness

Advanced imaging modalities, while invaluable for detecting both inflammatory and structural changes, can significantly increase trial costs. Balancing these costs with trial budgets without compromising the quality of data is a persistent challenge. Cost-effectiveness analyses should account for not only the financial implications but also the potential for imaging to improve patient outcomes by enabling more accurate assessments and treatment adjustments.

### Future research needs

The field of imaging axSpA continues to evolve, with several avenues for future research. Identifying novel imaging biomarkers that can predict disease progression or treatment response holds great promise [72]. Additionally, exploring new imaging modalities, such as ultra-high-field MRI or photon-counting CT, and evaluating their utility in axSpA trials could enhance our understanding of the disease and its management [73, 74].

### Artificial intelligence in axSpA clinical trials

Artificial intelligence (AI) is already being applied in clinical trials for axSpA, particularly in imaging analysis and predictive modeling. Advanced AI algorithms, such as deep learning models, have demonstrated high accuracy in identifying sacroiliitis on MRI and detecting structural changes in the SIJs and spine, often matching the performance of expert radiologists [75–77]. Similarly, machine learning techniques are being utilized to predict radiographic progression, aiding in patient stratification and individualized treatment planning [78]. However, the effectiveness of these tools depends on high-quality, standardized datasets, which can be challenging due to variability in imaging protocols and clinical assessments across trial centers. While AI shows promise for improving diagnostic consistency and streamlining trial processes, the costs and complexities of developing, validating, and deploying these algorithms remain significant barriers.

## Conclusion

In conclusion, imaging plays a pivotal role in advancing our understanding and management of axSpA, serving as both a diagnostic tool and a critical endpoint in clinical trials. While MRI and ld-CT have emerged as key modalities, challenges remain in standardizing protocols, ensuring cost-effectiveness, and optimizing longitudinal imaging for assessing treatment response and disease progression. The integration of AI holds promise for reducing the burden of image evaluation by improving efficiency and consistency in scoring. However, the potential for cost savings remains uncertain, given the substantial investments required to develop, validate, and deploy AI algorithms. Future research into novel biomarkers and imaging technologies promises to further enhance the precision and utility of imaging in axSpA, ultimately improving patient care and outcomes.

## Data Availability

Not applicable
